# Pharmacological profile of the aerial parts of *Rubus ulmifolius* Schott

**DOI:** 10.1186/s12906-017-1564-z

**Published:** 2017-01-19

**Authors:** Niaz Ali, Mohammad Shaoib, Syed Wadood Ali Shah, Ismail Shah, Muhammad Shuaib

**Affiliations:** 1 Department of Pharmacology, Institute of Basic Medical Sciences, Khyber Medical University, Peshawar, Khyber Pakhtunkhwa Pakistan; 2 Department of Pharmacy, University of Malakand, Chakdara, Dir Lower, Khyber Pakhtunkhwa Pakistan; 3 Department of Pharmacy, The Abdul Wali Khan University, Mardan, Kyber PakhtunKhwa Pakistan

**Keywords:** *Rubus ulmifolius*, Antipyretic, Paracetamol, Flavonoids, Brewer’s yeast, Pyrexia, Antioxidant

## Abstract

**Background:**

As aerial parts of *Rubus ulmifolius* contains phytochemicals like flavonoids and tannins. And whereas flavonoids and tannins have antioxidant and antipyretic activity, hence, current work is carried out to screen crude methanolic extract of aerial parts of *Rubus ulmifolius* (Ru.Cr) and crude flavonoids rich extract of *Rubus ulmifolius* (Ru.F) for possible antioxidant and antipyretic activity. Ru.Cr and Ru.F are also tested for brine shrimps lethality bioassay. Ru.F is tested for the first time for possible antioxidant and antipyretic activity.

**Methods:**

Preliminary phytochemical screening of Ru.Cr and Ru.F was performed as it provides rapid finger printing for targeting a pharmacological activity. Acute toxicity and Brine shrimps’ cytotoxicity studies of Ru.Cr and Ru.F were performed to determine its safe dose range. Antioxidant and antipyretic studies were also performed as per reported procedures.

**Results:**

Ru.Cr tested positive for presence of tannins, alkaloids, flavonoids and steroids. Ru.Cr is safe up to 6 g/kg following oral doses for acute toxicity study. Ru.Cr is safe up to 75 μg/kg (p.o), LC_50_ for Ru.Cr and Ru.F are 16.7 ± 1.4 μg/ml 10.6 ± 1.8 μg/ml, respectively (*n* = 3). Both Ru.Cr and Ru.F demonstrated comparable antioxidant activity using vitamin C as standard (*p* ≤ 0.05). In test dose of 300 mg of Ru.Cr, rectal temperature was reduced by 74% (*p* ≤ 0.05) on 4^th^ hour of the administration. More, Ru.F produced 72% reduction in pyrexia (*p* ≤ 0.05) on 4^th^ hour of administration of paracetamol in Westar rats.

**Conclusions:**

The current work confirms that aerial parts of *Rubus ulmifolius* contain flavonoids that are safe up to 6 g/kg (p.o). Crude methanolic extract and flavonoids rich fraction of *Rubus ulmifolius* have significant antioxidant and antipyretic activity. Further work is required to isolate the pharmacologically active substances for relatively safe and effective antipyretics and antioxidants.

## Background

Most of the people in world uses medicinal plants for treatment of different ailments as medicinal plants are rich source of drugs from natural sources [1]. The genus *Rubus* consists of more than 750 species and 12 subgenera. The genus is distributed all over the world, except Antarctica [2, 3]. Uses of *Rubus* species in folk medicine is based on its ethno-medicinal uses [4]. Different species of genius *Rubus* synthesize several varieties of medically active phytochemicals. Most of the phytochemicals are derivatives of phenolics, alkaloids, glycosides and terpenoids. Phenolics are most active biochemical constituents in species of *Rubus* [3]. Phenolics of *Rubus* include potent in vitro antioxidants, flavonoids, including compounds like flavanones, isoflavones, flavones, anthocyanins and catechins [5, 6]. Paste of roots of *Rubus ellipticus* is used to relieve severe headache [7]. In folk medicine, *Rubus idaeus* is used for treatment of colic pain and wounds in many countries. Extract of fruits of wild raspberry is used as diuretic [8]. Fruits of other species like *Rubus coreanus* is aphrodisiac, anti-inflammatory [9], anti-bacterial [10], antioxidant [11], anti-allergic [12] and anticancer [13] in traditional medicine. Traditionally, *Rubus fruticosus* has been in use for treatment of skin diseases, itching, scabies and eczema [14].

*Rubus ulmifolius* belongs to family Rosaceae [14]. In Italian traditional medicines, *Rubus ulmifolius*, has been in use for treatment of ulcers, abscesses, furuncles, red eyes, vaginal disorders, intestinal inflammations, diarrhea and hemorrhoids [15]. It is also used as antipyretic and carminative agent [16]. In Chilean folk medicine, *Rubus ulmifolius* is used for hypoglycemic activity [17]. It contains organic acids, ascorbic acid, volatile oils and tannins [18]. More, it has been reported that *Rubus ulmifolius* contains phenolics and flavonoids. Its key compounds are caffeic acid, ferulic acid, quercetin-3-O-glucuronide, kaempferol-3-*O*-β-d-glucuronide, gallic acid, ferulic acid and tiliroside [19]. Investigations for safe and effective herbal remedies for potent antipyretic activity gained momentum in recent as aspirin, paracetamol and nimesulide are toxic [20]. Thus safe and effective antipyretics are the need of time, preferably from plants’ kingdom. Flavonoids are found in different types of vegetables and fruits. Nuts, seeds, stems, flowers, tea, wine and honey are rich sources of flavonoids. Common constituents of chief dietary sources are flavonols, flavones, isoflavones, flavonones and biflavones [21–23]. Daily flavonoids intake ranges in 1–2 g/day [24]. On the other hand, flavonols and flavones daily intake was found as 23 mg, amongst which flavonol quercetin is 16 mg/day [25]. This represents that flavonoids, in general, are very safe. Flavonoids that demonstrate well defined structure activity relationships include flavones, flavanones, flavanonols, flavans, flavonols, catechins, anthocyanidins and isoflavone. Biological activities of the major flavonoids include anti-inflammatory, antioxidant, anticancer, antiviral and antibacterial. It has been reported that flavonoids have direct cyto-protective effect on coronary and vascular systems, the liver, and the pancreas. These distinctive characteristics of flavonoids place them as preferred phytochemicals amongst the natural products [26]. Rutin, quercetin (flavonols) and hesperidin (flavanone) have inhibited inflammation. Rutin is used in treatment of chronic phase of inflammatory arthritis [27]. Similarly, quercetin has been effective in reducing rat paw edema induced by carrageenan [28]. More, flavonoids have inhibited the enzymatic activity of cyclooxygenases and lipoxygenases that ultimately help as anti-inflammatory and antipyretic [29, 30] activity with decrease in inflammatory cytokines [31]. Flavonoids are very potent antioxidants against free radicals as well. They are described as free-radical scavengers like rutin and apigenin [32–35]. Free radical scavenging capacity is primarily accredited to high reactiveness of their hydroxyl substituents that take part in the reaction [25]. Due to their variety of pharmacological activities, flavonoids are referred as “nutraceuticals” [33, 36–38]. As *Rubus ulmifolius* is rich with flavonoids and phenolics that have antioxidant effects. In addition, rutin and are flavonoids that have been reported to have anti-inflammatory effects by preventing prostaglandins synthesis, hence, in the present investigations, an attempt has been made to evaluate pharmacological activities of aerial parts of *Rubus ulmifolius* on the basis of the reported phytochemical constituents like flavonoids. More, there are no reports for pharmacological screenings of total flavonoids of *Rubus ulmifolius*. Because of the potential antioxidant and antipyretic activity of flavonoids, we targeted *Rubus ulmifolius* for possible antipyretic activity. Acute toxicity and brine shrimp cytotoxicity of the extract as well as of the flavonoids are also performed to determine its safe dose range.

## Methods

### Plant materials

Fresh aerial parts of *Rubus ulmifolius* were collected in month of August 2012 from Chakdara (Dir Lower), Khyber Pakhtunkhwa Province of Pakistan. The plant was identified by Dr. Nasr Ullah Khan. A voucher specimen RU/023/013 was submitted to the herbarium of University of Malakand Chakdara, Dir Lower, Khyber Pakhtunkhwa.

### Preparation of crude extract

The collected aerial parts of *Rubus ulmifolius* were properly cleaned and subjected to shade drying. The dried plant materials (6.50 Kg) were mashed to fine powder using a mechanical grinder. The grinded materials were soaked in 11 liters of methanol (commercial grade) for 15 days. The soaked plant materials were frequently shaken and then filtered through Whatman Filter paper. The process was repeated three times. The filtrates were combined and concentrated using a rotary evaporator till a thick gummy and dark greenish crude extract (285.0 g) of *Rubus ulmifolius* (Ru.Cr) was obtained.

### Preparation of crude flavonoids rich extract

For extraction of crude flavonoids rich extract, shade dried materials of aerial parts of *Rubus ulmifolius* (180 g) were finely powdered through mesh size number 40. The crushed material was filled in thimbles and was made defatted through uninterrupted hot extraction by *n*-hexane. The menstruum was successively extracted with methanol using Soxhlet apparatus for 12 h till darkish green mass was obtained. The contents were concentrated through evaporation. It yielded 27 g of crude flavonoids rich extract *Rubus ulmifolius* (Ru.F).

### Animals

Albino mice (either sex) weighing in range 25–30 g were used in acute toxicity screening. Westar rats with average weight 180–200 g (either sex) were obtained from the animal house of University of Malakand, Khyber Pakhtunkhawa, Pakistan. Rats were used for screening of antipyretic activity. The animals were treated as per “Byelaws of the Scientific Procedures Issue-1 (2008) of University of Malakand”. The animals were kept in dry and clean cages on temperature 25 ± 3°C with 12h:12h dark/light cycle; relative humidity of 45–55%. The animals for the experiments were fasted overnight. However, they had free access to water. Ethical Committee of Department of Pharmacy, University of Malakand accorded approval as per “Byelaws of the Scientific Procedures Issue-1 (2008) of University of Malakand”.

### Statistics

All data are expressed as mean ± standard error of mean (SEM). Student’s *t*-test is applied using Graph Pad Prism with *p* < 0.05.

## Pharmacological activities

### Preliminary phytochemical screening

Ru.Cr was exposed to preliminary phytochemical examination for detection of terpenoids, flavonoids, glycosides, tannins, saponins, alkaloids, phenolic compounds, proteins, sterols and carbohydrates [39–41]. Similarly, Ru.F was also tested for flavonoids.

### Acute toxicity studies

Either sex Albino mice (weighing 25–30 g) were used for the evaluation of acute toxicity studies of Ru.Cr and Ru.F. Acute toxicity studies were conducted in two phases. Mice were fasted overnight. In phase 1, animals were allocated into three groups each having 3 mice. Ru.Cr was administered in test doses in 10, 100 and 1000 mg/kg. The animals were constantly observed for any behavioral alteration or death toll with follow up for 2 weeks. In 2^nd^ phase, additional three groups, all having 3 mice in each, received doses of 2000, 4000 and 6000 mg/kg of Ru.Cr per oral route, respectively. All animals were continuously observed for period of 2 weeks for behavioral changes and death toll.

Same procedure was used to determine the acute toxicity studies of Ru.F. Changes in behavior and death toll were recorded [42].

### Brine shrimp cytotoxicity study

Brine shrimp lethality bioassay was performed for assessment of cytotoxic activity of Ru.Cr and Ru.F. A plastic container which contained filtered artificial sea water already prepared by the dissolving 38.0 g of sea salt in 1 liter of distilled water was used in the experiments. Its pH was maintained on 8.5 that promotes the hatching of Brine shrimp (*Artemia salina*) eggs [43]. The container was incubated on room temperature for the next 48 h. After the hatching of eggs, live (larvae) nauplii which had got separated from the shells were obtained from the lightened part of compartment. Then, stock solutions of 10 mg/ml of both crude methanolic and the crude flavonoids extracts of *Rubus ulmifolius* were prepared by dissolving 50 mg of both the extracts (samples) in 5 ml distilled methanol each. From the stock solutions, different dilutions i.e. 2.5, 5, 10, 12.5, 25, 50, 75, 100, 250 and 500 μl were prepared in separate vials and left overnight to evaporate the solvent. Each vial (after solvent evaporation) was hosted with ten nauplii (10 brine shrimp larvae) through micro pipette. Volume in every vial was made with sea water up to 5 ml. All vials were maintained for 24 h at room temperature. Numbers of dead and survived shrimps were counted. The experiments were run in triplicate.

## Antioxidant activity

The antioxidant aspect of Ru.Cr and Ru.F were determined through % scavenging of 2,2-diphenyl-1-picrylhydrazyl (DPPH) free radical [44]. Ascorbic acid was dissolved in methanol to prepare 50 ml of stock solution in concentration of 500 ppm that served as standard antioxidant. In the same way, 50 ml of separate stock solutions were prepared for every test sample of Ru.Cr and Ru.F. Final concentrations of 40, 60, 80, 100 and 200 ppm (5 ml each test samples) were prepared in test tubes through serial dilutions. To each test tube, 1ml of stable DPPH was added. The test tubes were incubated in dark for 30 min at room temperature. Absorbances of test solutions were measured at 517 nm using UV spectrophotometer (Shimadzu UV-1700). Methanol was used as blank. Percent inhibition or free radical scavenging of DPPH was determined using following formula:

$$ \%\ \mathrm{inhibition}\ \mathrm{of}\ \mathrm{DPPH}=\frac{\mathrm{Absorbance}\ \mathrm{of}\ \mathrm{control}\hbox{-} \mathrm{Absorbance}\ \mathrm{of}\ \mathrm{sample}}{\mathrm{Absorbance}\ \mathrm{of}\ \mathrm{control}}\times 100 $$

### Antipyretic activity

Pyrexia was induced in rats by injecting 20% (20 g of dried Brewer’s yeast in 100 ml of 2% gum acacia and 0.9% saline aqueous suspension) of Brewer’s yeast in a dose of 1 ml/100 g body weight (s.c) [45]. The animals were fasted for 18 h. They had free access to water. Digital thermometer was used to record rectal temperature of test animals. Rats with a temperature rise of at least 0.5 °C to 1 °C were included in the study. Animals were divided into six groups (GI - GVI) having 6 rats in each group. The GI served as negative control group, which received 2% gum acacia (p.o). GII represented the standard group and received paracetamol (33 mg/kg of body weight in 2% gum acacia) (p.o). GIII and GIV received 150 mg/kg and 300 mg/kg body weight of Ru.Cr (p.o). While GV and GVI received 150 mg/kg and 300 mg/kg body weight of Ru.F (p.o), respectively. Rectal temperature of the animals was noted on an hour interval for 4 h following the administration of paracetamol and the test samples. Decrease in rectal body temperature of test animals was calculated by the following formula [46]:

$$ \mathrm{Percentage}\ \mathrm{reduction}\ \mathrm{in}\ \mathrm{pyrexia}=\frac{\mathrm{B}\hbox{-} \mathrm{C}n}{\mathrm{B}\hbox{-} \mathrm{A}}\times 100 $$

A: = Normal body temperature.
B: = Rectal temperature at 18^th^ h after yeast administration.
C*n*: = Rectal temperature after administration of drug.

## Results and discussion

### Preliminary phytochemical screening

Results for the presence of different phytochemicals are mentioned in Table 1. It tested positive for the presence of flavonoids, alkaloids, tannins, saponins, glycosides, phenolic compounds, sterols, carbohydrates, terpenoids and proteins.

**Table 1:** Results of different phytochemicals present in *Rubus ulmifolius*

S. No.	Phytochemicals	Results
1	Flavonoids	Positive
2	Alkaloids	Mild Positive
3	Tannins	Positive
4	Saponins	Positive
5	Proteins	Positive
6	Sterols	Positive
7	Carbohydrates	Positive
8	Glycosides	Positive
9	Terpenoids	Positive

### Acute toxicity studies

The purpose of acute toxicity studies is to determine a safe dose range which can be extrapolated to human model [47]. No death toll was observed till the maximum oral dose (6000 mg/kg) of Ru.Cr and Ru.F in all test groups. These results suggest that Ru.Cr and Ru.F are safe up to 6000 mg/kg body weight per oral route. Because of high margin of safety in acute toxicity study, we selected 150 mg/kg and 300 mg/kg oral doses for possible in vivo antipyretic activity.

### Brine shrimp cytotoxicity study

The effects of Ru.Cr and Ru.F on brine shrimps for possible lethality are shown in Table 2.

**Table 2:** Brine shrimp cytotoxicity assay of test samples of *Rubus ulmifolius*

Conc. (μg/ml)	Number of Brine shrimps	Brine shrimps killed (Mean ± SD, *n* = 3)	
		Ru.Cr	Ru.F
Negative Control	10	00	00
2.5	10	3 ± 0.47	3.23 ± 0.27
5	10	3.33 ± 0.27	4.11 ± 0.27
10	10	4 ± 0 .47*	5 ± 0 .47**
12.5	10	5 ± 0.27**	7.33 ± 0.54***
25	10	6.36 ± 0.27**	9.33 ± 0.27***
50	10	7.42 ± 0.27***	9.66 ± 0.27***
75	10	9.33 ± 0.27	All dead
100	10	All dead	All dead
250	10	All dead	All dead
500	10	All dead	All dead
LC_50_ (μg/ml)		16.7 ± 1.4	10.6 ± 1.8

All values are taken as mean ± SD, **P < 0.05, ***P < 0.01

The magnitude of lethality was directly proportional to the concentration of test samples. Ru.Cr has an LC_50_ of 16.7 ± 1.4 μg/ml. LC_max∞100%_ was in concentration of 100 μg/ml. Similarly, Ru.F has an LC_50_ of 10.6 ± 1.8 μg/ml with maximum mortality in concentration of 75 μg/ml i.e. LC_max∞100%_. This explains that the flavonoids rich extract may have cytotoxic constituents as well. This study justifies that the plant may be a potential source for isolation of anticancer bioactive molecules as there are reports that correlate simple bench‐top bioassays and human tumour cell cytotoxicities as potential antitumor agents [48, 49]. This needs for isolation of bioactive molecules as plants are good sources of anticancer drugs.

### Antioxidant activity

Results of antioxidant activity of Ru.Cr and Ru.F are shown in Table 3.

**Table 3:** Antioxidant activity of *Rubus ulmifolius*

Conc. (ppm)	% scavenging of stable DPPH free radical		
	Ascorbic acid (Control)	Ru.Cr	Ru.F
40	62.70 ± 0.291	59.4 ± 0.073***	61.43 ± 0.256***
60	69.96 ± 0.522	61.96 ± 0.259***	68.61 ± 0.262***
80	75.03 ± 0.328	66.10 ± 0.275**	73.20 ± 0.233***
100	81.26 ± 0.233	71.26 ± 0.321***	79.38 ± 0.298***
200	87.26 ± 0.128	74.2 ± 0.324***	83.16 ± 0.091***

All values are taken as mean ± SEM (*n* = 3), **P < 0.05, ***P < 0.01

Ru.Cr showed 59.4 ± 0.073% inhibition in concentration of 40 ppm and 74.2 ± 0.324% inhibition in concentration of 200 ppm. Percent scavenging activity of ascorbic acid was 87.26 ± 0.128%. Free radical scavenging with Ru.F is 61.43 ± 0.256% in concentration of 40 ppm. Ascorbic acid inhibited the free radical scavenging activity up to 62.7 ± 0.291%. More, in highest concentration (200 ppm), Ru.F showed 83.16 ± 0.091% free radical scavenging activity. While ascorbic acid demonstrated 87.26 ± 0.128% free radical scavenging activity. This shows that the free radical scavenging activity of Ru.F is comparable with ascorbic acid, a standard antioxidant.

In basic process of oxidation, the most important step is the formation of free radicals (having unpaired electrons) in living systems, drugs and even food [50]. Free radicals, which are extremely unstable, causes many diseases of kidney and liver, Alzheimer’s disease, fibrosis, cardiac reperfusion abnormalities, atherosclerosis, cancer, arthritis, neurodegenerative disorders and aging [51].

It is reported that antioxidants prevent the oxidative damage which is caused by reactive oxygen species (ROS) and free radicals [52]. Antioxidants act by interfering in the oxidation process reaction with the chelating, catalytic metals and free radicals as oxygen scavengers [53, 54]. Ru.Cr and Ru.F produced comparable free radical scavenging activities both in low and high concentrations versus standard ascorbic acid. The order of free radical scavenging of test samples (40 and 200ppm) and standard is: ascorbic acid > Ru. F > Ru.Cr. Our findings confirm reports that *Rubus* species are extremely abundant with phenolic contents like flavonoids, phenolic acids and anthocyanins. These phenolic compounds have been reported to possess antioxidant, anti-inflammatory and anticancer properties [55]. Amongst different species of genus *Rubus, Rubus ellipticus* has exhibited antioxidant activities [56]. *Rubus coreanus* is reported to have antioxidant properties [57]. It has been reported that the phenolic compounds like flavonoids exhibited prominent antioxidant activities [58]. Based on reports for importance of phenolic components as antioxidants, the observed antioxidant activity may be attributed to the occurrence of flavonoids like active constituents which require further research for isolation of antioxidant components from the *Rubus ulmifolius*.

### Antipyretic studies

Both Ru.Cr and Ru.F demonstrated significant antipyretic as mentioned in Table 4. Antipyretic effects are comparable with antipyretic effects of paracetamol, a standard antipyretic agent.

**Table 4:** Effect of crude and flavonoids extracts of *Rubus ulmifolius* on yeast-induced pyrexia in Rats

Groups		Rectal Temperature in °C					
		Normal	Brewer’s yeast	After administration of test samples and standard drug			
				1^st^hr	2^nd^hr	3^rd^hr	4^th^hr
GI (Control)		37.36 ± 0.08	39.61 ± 0.03	39.41 ± 0.04 (8.88%)	39.48 ± 0.06 (5.92%)	39.43 ± 0.07 (8.14%)	39.46 ± 0.04 (6.66%)
GII (Paracetamol)		37.46 ± 0.04	39.4 ± 0.06	38.86 ± 0.08 (27.58%)	38.38 ± 0.07 (52.58%)	38 ± 0.05*** (72.41%)	37.7 ± 0.05*** (87.93%)
Ru.Cr	GIII 150 mg	37.13 ± 0.03	39.45 ± 0.24	39.36 ± 0.08 (3.87%)	39.10 ± 0.15 (15.08%)	38.76 ± 0.23 (29.74%)	38.20 ± 0.11* (53.87%)
	GIV 300 mg	37.23 ± 0.08	38.90 ± 0.30	38.71 ± 0.08 (11.37%)	38.36 ± 0.34 (32.33%)	37.93 ± 0.42** (58.08%)	37.66 ± 0.38*** (74.25%)
Ru.F	GV 150 mg	37.16 ± 0.08	39.36 ± 0.38	38.84 ± 0.12 (23.63%)	38.71 ± 0.35 (29.54%)	38.53 ± 0.12 (37.72%)	37.87 ± 0.50** (67.72%)
	GVI 300 mg	37.26 ± 0.12	39.66 ± 0.20	38.70 ± 0.45 (40%)	38.23 ± 0.08** (59.58%)	37.86 ± 0.18*** (75%)	37.60 ± 0.05*** (85.83%)

All values are taken as mean ± SEM (*n* = 3), **P < 0.05, ***P < 0.01

Ru.Cr in test dose of 300 mg reduced rectal temperature up to 74% on 4^th^ hour of the administration. On 3^rd^ hour, Ru.Cr decreased body temperature by 58%. Similarly, Ru.F produced 72% reduction in body temperature on 4^th^ hour of the administration. Following 2^nd^ hour of administration of Ru.F, decrease in body temperature was 59%, which is 52% of paracetamol, a standard antipyretic. This suggests that Ru.Cr and Ru.F have antipyretic activity. Antipyretic drugs are used to reduce the elevated body temperature both by depression of inflammatory mediators at sites of peripheral tissues, and at thermoregulatory sites within the Central Nervous System (CNS) [59]. NSAIDs are having antipyretic action due to their inhibitory effects on cyclooxygenase (COX), an enzyme responsible for production of prostaglandins, which are important mediators for producing pain and pyrexia. NSAIDs are said to possess antipyretic properties which resets the hypothalamic thermostat and thus reduces elevated body temperature during fever. Heat loss is promoted by cutaneous vasodilatation and sweating. Paracetamol produces anti-pyretic action while acting on CNS [60].

Yeast administration induces the release of phagocytes and interleukins (endogenous Pyrogens). Interleukins helps the production of T-lymphocytes, which in turn induce - hypothalamus to produce prostaglandins that finally elevate the body temperature [61].

In a test dose of 300 mg, it is evident that Ru.F produced 85.83% fall in body temperature. These results confirm that the crude methanolic extract and crude flavonoids rich extract of *Rubus ulmifolius* have significant antipyretic effect. This significant antipyretic effect of *Rubus ulmifolius* is attributed to the presence of phytochemicals like flavonoids, which are reported for inhibitory effects on cyclooxygenases [62, 63]. The flavonoids are known to act by blocking the synthesis of prostaglandins E2 predominantly through inhibition of prostaglandins synthetase enzyme [64]. Hence, it is deduced that the antipyretic effects may be attributed to flavonoids present in the species.

Thus, the present study confirms the folkloric use of *Rubus ulmifolius* as antipyretic remedy. However, further studies are suggested to isolate the pharmacologically active antipyretic constituents from the species.

## Conclusions

The current work confirms that aerial parts of *Rubus ulmifolius* contain flavonoids that are safe up to 6g/kg (p.o). Crude methanolic extract and flavonoids rich fraction of *Rubus ulmifolius* have significant antioxidant and antipyretic activity. Further work is required to isolate the pharmacologically active substances for relatively safe and effective antipyretics and antioxidants.

## Abbreviations

LC_50_: Least concentration of a test sample that kills 50% of the nauplii
LC_max∞100%_: Least concentration of test sample that kills 100% of the nauplii. P.O per oral
Ru.Cr: Crude methanolic extract of *Rubus ulmifolius*
Ru.F: Extract of crude flavonoids rich fraction of *Rubus ulmifolius*
S.C: Sub cutaneous
